# The genome sequence of the Arctic Skipper, *Carterocephalus palaemon *(Pallas, 1771)

**DOI:** 10.12688/wellcomeopenres.19573.1

**Published:** 2023-08-30

**Authors:** Konrad Lohse, Sam Ebdon, Alex Mackintosh, Simon Martin, Ilik J Saccheri, Nigel A D Bourn, Roger Vila, Wellcome Sanger Institute Tree of Life programme, Wellcome Sanger Institute Scientific Operations: DNA Pipelines collective, Tree of Life Core Informatics collective, Darwin Tree of Life Consortium

**Affiliations:** 1 Institute of Ecology and Evolution, The University of Edinburgh, Edinburgh, Scotland, UK; 2 Department of Evolution, Ecology and Behaviour,, University of Liverpool, Liverpool, England, UK; 3 Butterfly Conservation, Wareham, England, UK; 4 Institut de Biologia Evolutiva (CSIC - Universitat Pompeu Fabra), Barcelona, Spain

**Keywords:** Carterocephalus palaemon, Arctic Skipper, genome sequence, chromosomal, Lepidoptera

## Abstract

We present a genome assembly from an individual male *Carterocephalus palaemon* (the Arctic Skipper; Arthropoda; Insecta; Lepidoptera; Hesperiidae). The genome sequence is 394.5 megabases in span. The whole assembly is scaffolded into 31 chromosomal pseudomolecules, including the Z sex chromosome. The mitochondrial genome has also been assembled and is 15.78 kilobases in length. Gene annotation of this assembly on Ensembl identified 17,032 protein coding genes.

## Species taxonomy

Eukaryota; Metazoa; Eumetazoa; Bilateria; Protostomia; Ecdysozoa; Panarthropoda; Arthropoda; Mandibulata; Pancrustacea; Hexapoda; Insecta; Dicondylia; Pterygota; Neoptera; Endopterygota; Amphiesmenoptera; Lepidoptera; Glossata; Neolepidoptera; Heteroneura; Ditrysia; Obtectomera; Hesperioidea; Hesperiidae; Heteropterinae; *Carterocephalus*; *Carterocephalus palaemon* (Pallas, 1771) (NCBI:txid218720).

## Background

The Chequered Skipper (also known as the Arctic Skipper), *Carterocephalus palaemon* (Pallas, 1771), is a small butterfly that flies in rapid zigzagging dashes, inhabiting woodland glades or edges and wet meadows. It has a broadly Holarctic distribution that is often patchy, with relatively low local abundances. Phylogenomic analysis ( Zhang *et al.*, 2021) supports the distinction of three subspecies (or species): *C. palaemon palaemon* (Eurasia); *C. palaemon skada* (northern North America); and *C. palaemon magnus* (western North America). The Chequered Skipper is listed as a species of Least Concern both on the IUCN Red List (Europe) ( Van Swaay *et al.*, 2010) and the GB Red List ( Fox *et al.*, 2022).

In Britain, until its reintroduction into England starting in 2018, the species was restricted to an isolated population in western Scotland (source for the DToL genome assembly), having gone extinct from England in 1976 ( Asher *et al.*, 2001), owing to the decline in coppicing and other changes to forestry practice.

Although mitochondrial DNA evidence suggests that the historical English population is closely related to the Scottish one ( Joyce & Pullin, 2004), the source material used for the reintroduction into Rockingham Forest (Northamptonshire) came from Belgian sites with similar ecological conditions ( Maes *et al.*, 2019). In Scotland, the main host plant is Purple Moor-grass ( *Molinia caerulea*), whereas in England it is Wood Small-reed ( *Calamagrostis epigejos*) and False-brome ( *Brachypodium sylvaticum*).

The karyotype of the Chequered Skipper is unknown. The Darwin Tree of Life reference genome we present here will facilitate non-invasive monitoring of genetic variability and ancestry in the reintroduced population.

## Genome sequence report

**Figure 1.**
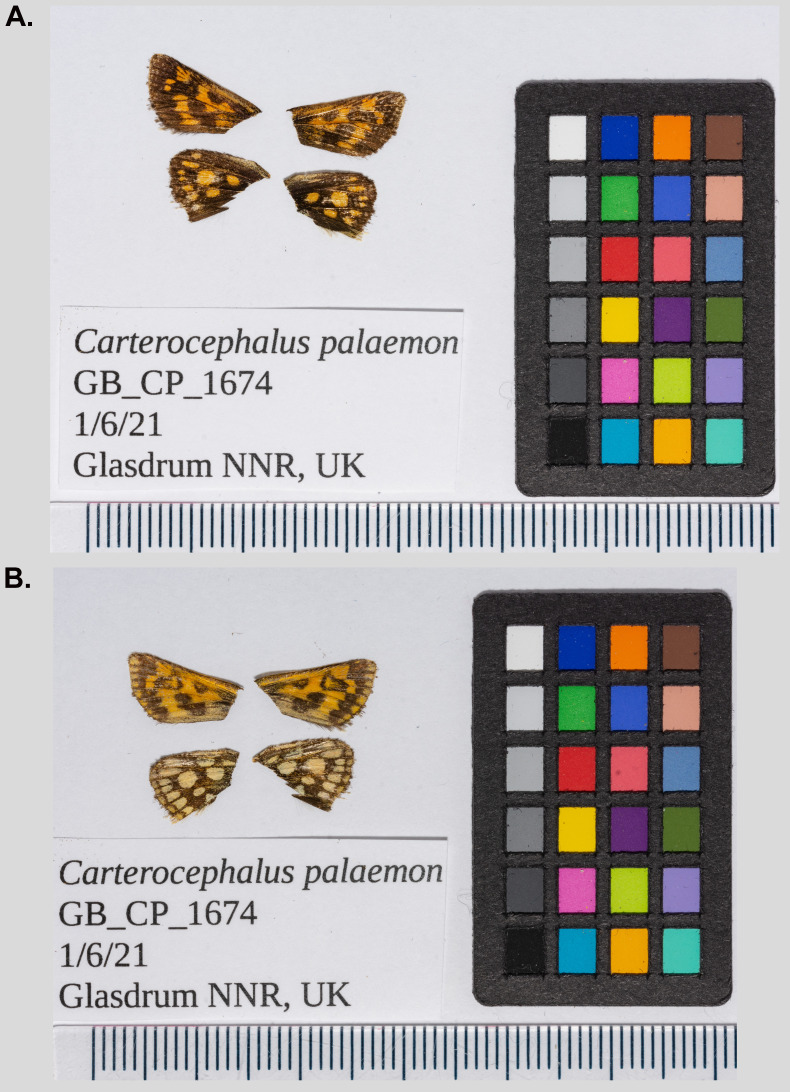
Dorsal ( **A**) and ventral ( **B**) surface view of wings from specimen GB_CP_1674 (ilCarPala2) from Glasdrum National Nature Reserve, Scotland.

The genome was sequenced from one male *Carterocephalus palaemon* ( Figure 1) collected from Glasdrum National Nature Reserve, Scotland (56.57, –5.23). A total of 45-fold coverage in Pacific Biosciences single-molecule HiFi long reads was generated. Primary assembly contigs were scaffolded with chromosome conformation Hi-C data. Manual assembly curation corrected 16 missing joins or mis-joins and removed one haplotypic duplication, reducing the scaffold number by 20%.

**Figure 2.**
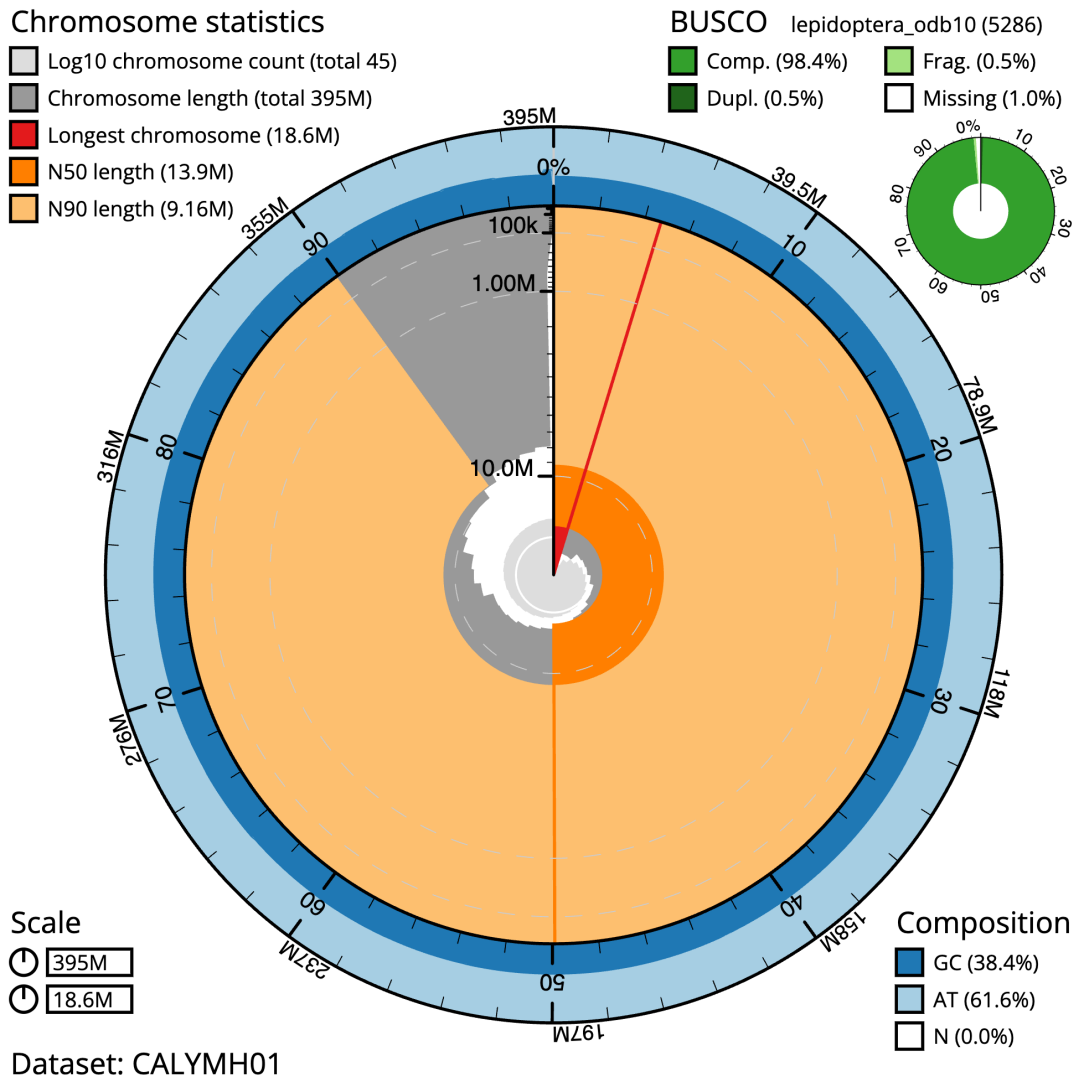
Genome assembly of *Carterocephalus palaemon*, ilCarPala2.1: metrics. The BlobToolKit Snailplot shows N50 metrics and BUSCO gene completeness. The main plot is divided into 1,000 size-ordered bins around the circumference with each bin representing 0.1% of the 394,538,208 bp assembly. The distribution of scaffold lengths is shown in dark grey with the plot radius scaled to the longest scaffold present in the assembly (18,628,966 bp, shown in red). Orange and pale-orange arcs show the N50 and N90 scaffold lengths (13,897,380 and 9,164,508 bp), respectively. The pale grey spiral shows the cumulative scaffold count on a log scale with white scale lines showing successive orders of magnitude. The blue and pale-blue area around the outside of the plot shows the distribution of GC, AT and N percentages in the same bins as the inner plot. A summary of complete, fragmented, duplicated and missing BUSCO genes in the lepidoptera_odb10 set is shown in the top right. An interactive version of this figure is available at https://blobtoolkit.genomehubs.org/view/ilCarPala2.1/dataset/CALYMH01/snail.

The final assembly has a total length of 394.5 Mb in 44 sequence scaffolds with a scaffold N50 of 13.9 Mb ( Table 1). The whole assembly sequence was assigned to 31 chromosomal-level scaffolds, representing 30 autosomes and the Z sex chromosome. Chromosome-scale scaffolds confirmed by the Hi-C data are named in order of size ( Figure 2– Figure 5; Table 2). While not fully phased, the assembly deposited is of one haplotype. Contigs corresponding to the second haplotype have also been deposited. The mitochondrial genome was also assembled and can be found as a contig within the multifasta file of the genome submission.

**Table 1.** Genome data for *Carterocephalus palaemon*, ilCarPala2.1.

Project accession data		
Assembly identifier	ilCarPala2.1	
Species	*Carterocephalus palaemon*	
Specimen	ilCarPala2	
NCBI taxonomy ID	218720	
BioProject	PRJEB52799	
BioSample ID	SAMEA9700885	
Isolate information	ilCarPala2, male: whole organism (DNA sequencing and Hi-C scaffolding)	
Assembly metrics *		*Benchmark*
Consensus quality (QV)	66.3	*≥ 50*
*k*-mer completeness	100%	*≥ 95%*
BUSCO **	C:98.4%[S:97.9%,D:0.5%], F:0.5%,M:1.0%,n:5,286	*C ≥ 95%*
Percentage of assembly mapped to chromosomes	100%	*≥ 95%*
Sex chromosomes	Z chromosome	*localised homologous* *pairs*
Organelles	Mitochondrial genome assembled	*complete single alleles*
Raw data accessions		
PacificBiosciences SEQUEL II	ERR9793196	
Hi-C Illumina	ERR9730868	
Genome assembly		
Assembly accession	GCA_944567765.1	
*Accession of alternate* *haplotype*	GCA_944567795.1	
Span (Mb)	394.5	
Number of contigs	61	
Contig N50 length (Mb)	13.1	
Number of scaffolds	44	
Scaffold N50 length (Mb)	13.9	
Longest scaffold (Mb)	18.6	
Genome annotation		
Number of protein- coding genes	17,032	
Number of gene transcripts	17,333	

* Assembly metric benchmarks are adapted from column VGP-2020 of “Table 1: Proposed standards and metrics for defining genome assembly quality” from ( Rhie *et al.*, 2021). ** BUSCO scores based on the lepidoptera_odb10 BUSCO set using v5.3.2. C = complete [S = single copy, D = duplicated], F = fragmented, M = missing, n = number of orthologues in comparison. A full set of BUSCO scores is available at https://blobtoolkit.genomehubs.org/view/ilCarPala2.1/dataset/CALYMH01/busco.

**Figure 3.**
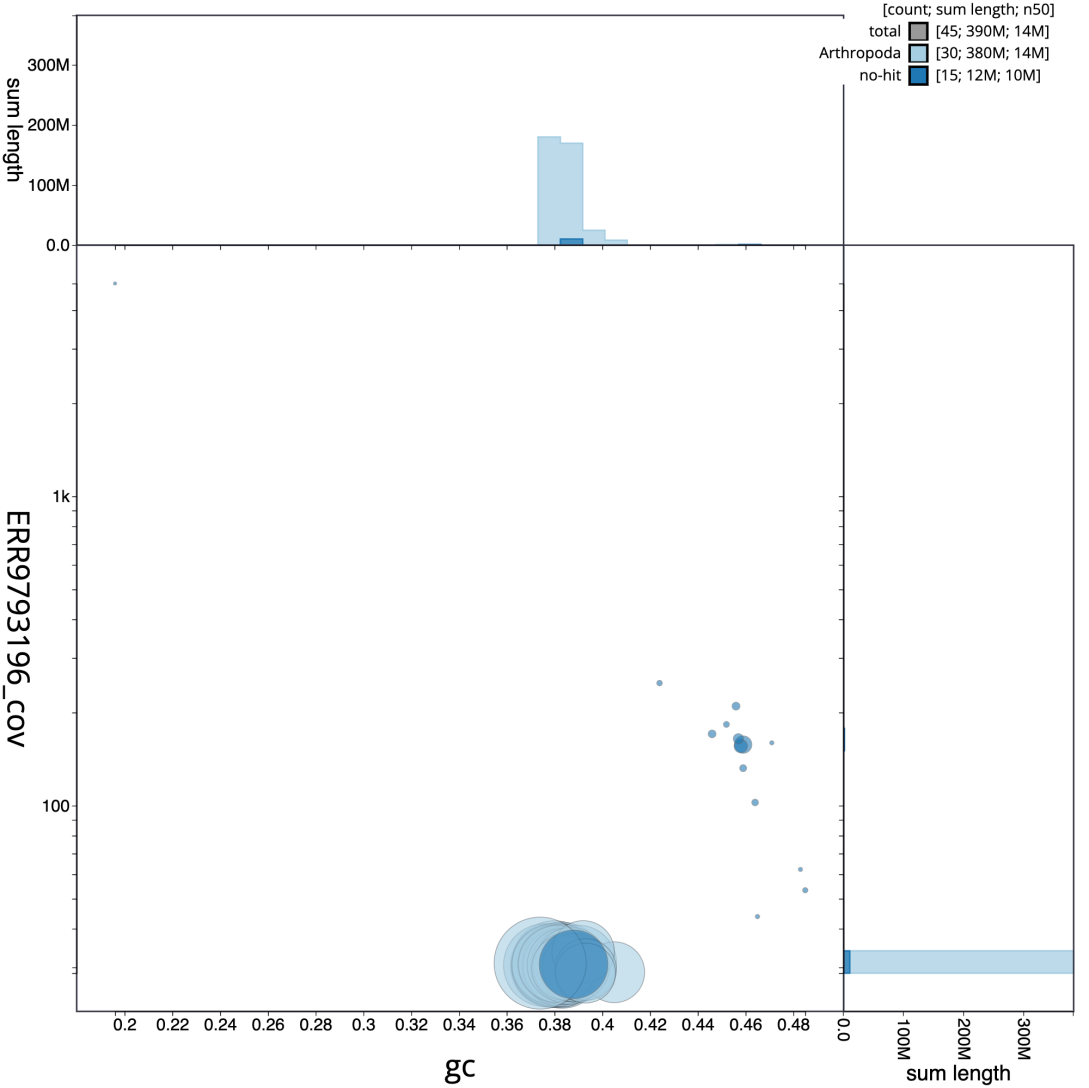
Genome assembly of *Carterocephalus palaemon*, ilCarPala2.1: BlobToolKit GC-coverage plot. Scaffolds are coloured by phylum. Circles are sized in proportion to scaffold length. Histograms show the distribution of scaffold length sum along each axis. An interactive version of this figure is available at https://blobtoolkit.genomehubs.org/view/ilCarPala2.1/dataset/CALYMH01/blob.

**Figure 4.**
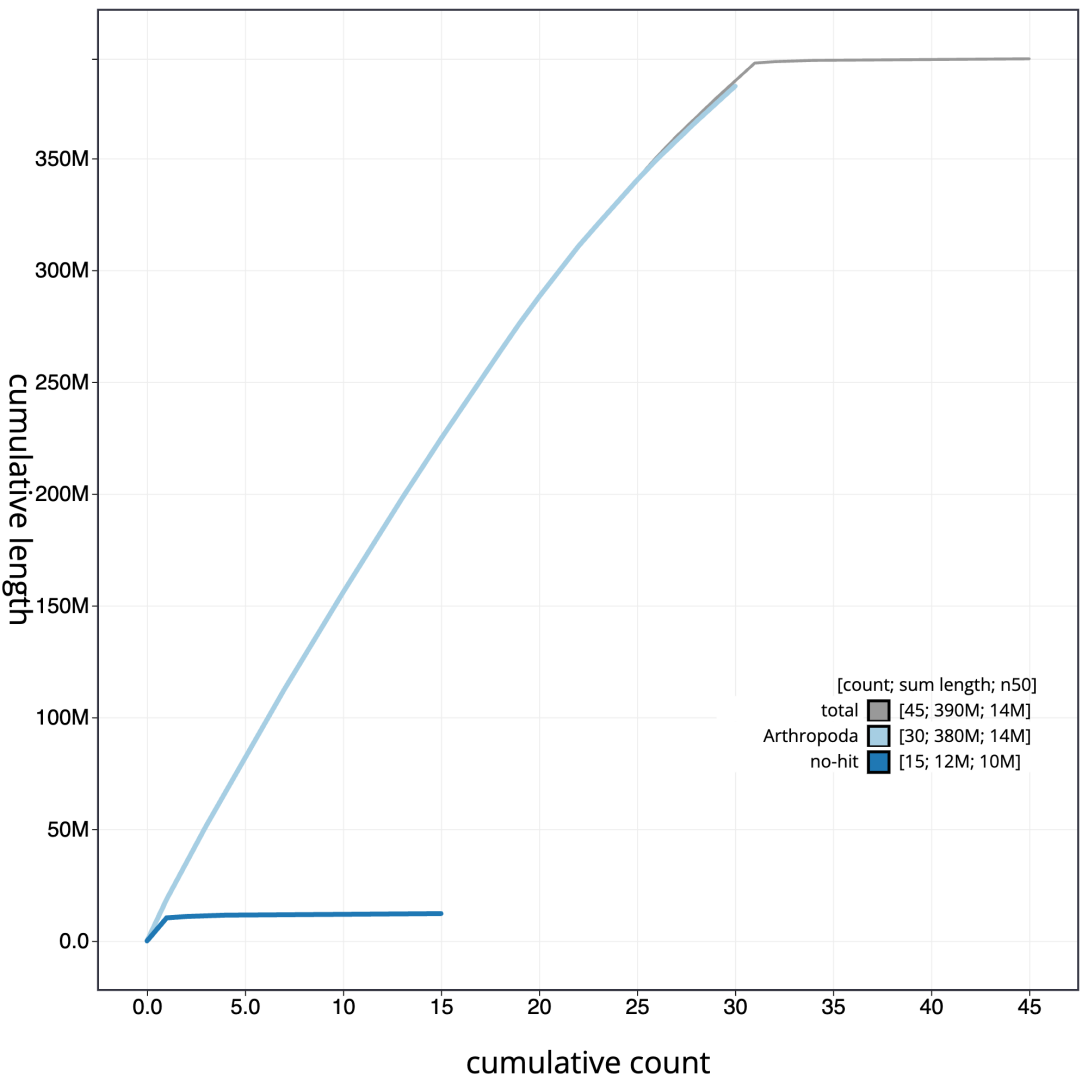
Genome assembly of *Carterocephalus palaemon*, ilCarPala2.1: BlobToolKit cumulative sequence plot. The grey line shows cumulative length for all scaffolds. Coloured lines show cumulative lengths of scaffolds assigned to each phylum using the buscogenes taxrule. An interactive version of this figure is available at https://blobtoolkit.genomehubs.org/view/ilCarPala2.1/dataset/CALYMH01/cumulative.

**Figure 5.**
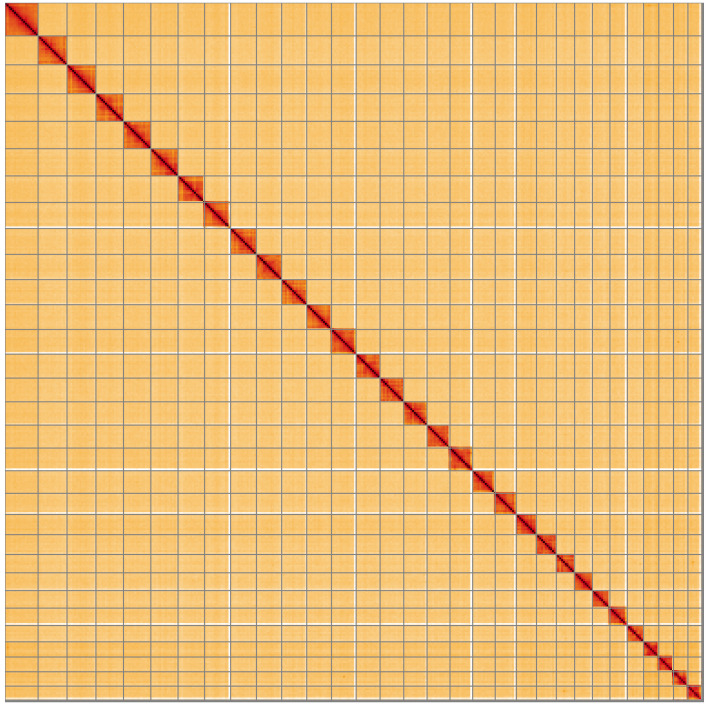
Genome assembly of *Carterocephalus palaemon*, ilCarPala2.1: Hi-C contact map of the ilCarPala2.1 assembly, visualised using HiGlass. Chromosomes are shown in order of size from left to right and top to bottom. An interactive version of this figure may be viewed at https://genome-note-higlass.tol.sanger.ac.uk/l/?d=GepxHsyRR8-FnB77oidXAw.

**Table 2.** Chromosomal pseudomolecules in the genome assembly of *Carterocephalus palaemon*, ilCarPala2.

INSDC accession	Chromosome	Length (Mb)	GC%
OX155638.1	1	16.31	38.0
OX155639.1	2	16.29	38.5
OX155640.1	3	15.54	38.0
OX155641.1	4	15.41	38.0
OX155642.1	5	15.3	38.0
OX155643.1	6	14.99	37.5
OX155644.1	7	14.61	38.0
OX155645.1	8	14.57	38.0
OX155646.1	9	14.25	38.0
OX155647.1	10	14.09	38.0
OX155648.1	11	13.94	38.5
OX155649.1	12	13.9	38.5
OX155650.1	13	13.49	38.5
OX155651.1	14	13.35	38.0
OX155652.1	15	13.13	38.5
OX155653.1	16	12.92	38.0
OX155654.1	17	12.78	39.0
OX155655.1	18	12.71	38.5
OX155656.1	19	11.89	38.5
OX155657.1	20	11.36	39.0
OX155658.1	21	11.16	38.5
OX155659.1	22	10.32	39.0
OX155660.1	23	10.04	39.0
OX155661.1	24	8.11	40.5
OX155662.1	25	9.83	38.5
OX155663.1	26	9.82	39.0
OX155664.1	27	9.16	38.5
OX155665.1	28	8.54	39.0
OX155666.1	29	8.31	39.5
OX155667.1	30	7.85	39.5
OX155637.1	Z	18.63	37.5
OX155668.1	MT	0.02	19.5

The estimated Quality Value (QV) of the final assembly is 66.3 with *k*-mer completeness of 100%, and the assembly has a BUSCO v5.3.2 completeness of 98.4% (single = 97.9%, duplicated = 0.5%), using the lepidoptera_odb10 reference set ( *n* = 5,286).

Metadata for specimens, spectral estimates, sequencing runs, contaminants and pre-curation assembly statistics can be found at https://links.tol.sanger.ac.uk/species/218720.

## Genome annotation report

The *Carterocephalus palaemon* genome assembly (GCA_944567765.1) was annotated using the Ensembl rapid annotation pipeline ( Table 1; https://rapid.ensembl.org/Carterocephalus_palaemon_GCA_944567765.1/Info/Index). The resulting annotation includes 17,333 transcribed mRNAs from 17,032 protein-coding genes.

## Methods

### Sample acquisition and nucleic acid extraction

The specimen used for genome sequencing was a male *Carterocephalus palaemon* (ilCarPala2), hand netted in Glasdrum National Nature Reserve, Scotland, UK (latitude 56.57, longitude –5.23) on 2021-06-01. The collectors were Konrad Lohse, Sam Ebdon, Alex Mackintosh and Simon Martin (all University of Edinburgh). The specimen was identified by Konrad Lohse and then snap-frozen from live in a dry shipper.

DNA was extracted at the Tree of Life laboratory, Wellcome Sanger Institute (WSI). The ilCarPala2 sample was weighed and dissected on dry ice with tissue set aside for Hi-C sequencing. Whole organism tissue was disrupted using a Nippi Powermasher fitted with a BioMasher pestle. High molecular weight (HMW) DNA was extracted using the Qiagen MagAttract HMW DNA extraction kit. HMW DNA was sheared into an average fragment size of 12–20 kb in a Megaruptor 3 system with speed setting 30. Sheared DNA was purified by solid-phase reversible immobilisation using AMPure PB beads with a 1.8X ratio of beads to sample to remove the shorter fragments and concentrate the DNA sample. The concentration of the sheared and purified DNA was assessed using a Nanodrop spectrophotometer and Qubit Fluorometer and Qubit dsDNA High Sensitivity Assay kit. Fragment size distribution was evaluated by running the sample on the FemtoPulse system.

### Sequencing

Pacific Biosciences HiFi circular consensus DNA sequencing libraries were constructed according to the manufacturers’ instructions. DNA sequencing was performed by the Scientific Operations core at the WSI on the Pacific Biosciences SEQUEL II (HiFi) instrument. Hi-C data were also generated from tissue of ilCarPala2 using the Arima2 kit and sequenced on the Illumina NovaSeq 6000 instrument.

### Genome assembly, curation and evaluation

Assembly was carried out with Hifiasm ( Cheng *et al.*, 2021) and haplotypic duplication was identified and removed with purge_dups ( Guan *et al.*, 2020). The assembly was then scaffolded with Hi-C data ( Rao *et al.*, 2014) using YaHS ( Zhou *et al.*, 2023). The assembly was checked for contamination and corrected using the gEVAL system ( Chow *et al.*, 2016) as described previously ( Howe *et al.*, 2021). Manual curation was performed using gEVAL, HiGlass ( Kerpedjiev *et al.*, 2018) and Pretext ( Harry, 2022). The mitochondrial genome was assembled using MitoHiFi ( Uliano-Silva *et al.*, 2022), which runs MitoFinder ( Allio *et al.*, 2020) or MITOS ( Bernt *et al.*, 2013) and uses these annotations to select the final mitochondrial contig and to ensure the general quality of the sequence.

A Hi-C map for the final assembly was produced using bwa-mem2 ( Vasimuddin *et al.*, 2019) in the Cooler file format ( Abdennur & Mirny, 2020). To assess the assembly metrics, the *k*-mer completeness and QV consensus quality values were calculated in Merqury ( Rhie *et al.*, 2020). This work was done using Nextflow ( Di Tommaso *et al.*, 2017) DSL2 pipelines “sanger-tol/readmapping” ( Surana *et al.*, 2023) and “sanger-tol/genomenote” ( Surana *et al.,* 2023). The genome was analysed within the BlobToolKit environment ( Challis *et al.*, 2020) and BUSCO scores ( Manni *et al.*, 2021; Simão *et al.*, 2015) were calculated.

Table 3 contains a list of relevant software tool versions and sources.

**Table 3.** Software tools: versions and sources.

Software tool	Version	Source
BlobToolKit	4.0.7	https://github.com/blobtoolkit/blobtoolkit
BUSCO	5.3.2	https://gitlab.com/ezlab/busco
gEVAL	N/A	https://geval.org.uk/
Hifiasm	0.16.1-r375	https://github.com/chhylp123/hifiasm
HiGlass	1.11.6	https://github.com/higlass/higlass
Merqury	MerquryFK	https://github.com/thegenemyers/MERQURY.FK
MitoHiFi	2	https://github.com/marcelauliano/MitoHiFi
PretextView	0.2	https://github.com/wtsi-hpag/PretextView
purge_dups	1.2.3	https://github.com/dfguan/purge_dups
sanger-tol/genomenote	v1.0	https://github.com/sanger-tol/genomenote
sanger-tol/readmapping	1.1.0	https://github.com/sanger-tol/readmapping/tree/1.1.0
YaHS	yahs-1.1.91eebc2	https://github.com/c-zhou/yahs

### Genome annotation

The BRAKER2 pipeline ( Brůna *et al.*, 2021) was used in the default protein mode to generate annotation for the *Carterocephalus palaemon* assembly (GCA_944567765.1) in Ensembl Rapid Release.

### Wellcome Sanger Institute – Legal and Governance

The materials that have contributed to this genome note have been supplied by a Tree of Life collaborator. The Wellcome Sanger Institute employs a process whereby due diligence is carried out proportionate to the nature of the materials themselves, and the circumstances under which they have been/are to be collected and provided for use. The purpose of this is to address and mitigate any potential legal and/or ethical implications of receipt and use of the materials as part of the research project, and to ensure that in doing so we align with best practice wherever possible. The overarching areas of consideration are:

- Ethical review of provenance and sourcing of the material
- Legality of collection, transfer and use (national and international)

Each transfer of samples is undertaken according to a Research Collaboration Agreement or Material Transfer Agreement entered into by the Tree of Life collaborator, Genome Research Limited (operating as the Wellcome Sanger Institute) and in some circumstances other Tree of Life collaborators.

## Data Availability

European Nucleotide Archive: *Carterocephalus palaemon* (Arctic skipper). Accession number PRJEB52799; https://identifiers.org/ena.embl/PRJEB52799. ( Wellcome Sanger Institute, 2022) The genome sequence is released openly for reuse. The *Carterocephalus palaemon* genome sequencing initiative is part of the Darwin Tree of Life (DToL) project. All raw sequence data and the assembly have been deposited in INSDC databases. Raw data and assembly accession identifiers are reported in Table 1.

## Author information

Members of the Wellcome Sanger Institute Tree of Life programme are listed here: https://doi.org/10.5281/zenodo.4783585.

Members of Wellcome Sanger Institute Scientific Operations: DNA Pipelines collective are listed here: https://doi.org/10.5281/zenodo.4790455.

Members of the Tree of Life Core Informatics collective are listed here: https://doi.org/10.5281/zenodo.5013541.

Members of the Darwin Tree of Life Consortium are listed here: https://doi.org/10.5281/zenodo.4783558.
