# Supplementary material for: The genome sequence of the Arctic Skipper, Carterocephalus palaemon (Pallas, 1771)
Source: Wellcome Open Res. 2023 Aug 30;8:369. [Version 1] doi: 10.12688/wellcomeopenres.19573.1 (PMC11303956; doi:10.12688/wellcomeopenres.19573.1)
Supplement: Peer review history

# Reviewer report: Reviewer response for version 1

**DOI:** 10.21956/wellcomeopenres.21683.r89255

**Published:** 2024-08-06

**Authors:** Jerome H L Hui

**Affiliations:** 1 The Chinese University of Hong Kong, Hong Kong, Hong Kong

In this Data Note, Lohse and colleagues reported the genome assembly of a male butterfly *Carterocephalus palaemon *(Pallas, 1771), which is also commonly known as the chequered skipper or arctic skipper. According to the UK BAP, it is protected under Schedule 5 of the Wildlife and Countryside Act 1981, with respect to sale only. Molecular data of this species are limited to few marker genes deposited to the NCBI database. Therefore, this new genome resource will be useful for further studies into many areas, including but not limited to understanding its population structure, ecology, insect-plant interaction, and evolution with other insects more widely. In addition, given it is a priority species listed in the UK BAP, the high-quality genomic resource provided in this study will also be useful for designing and carrying out its different conservation measures.

 This genome resource is excellent from the summary statistics, with high BUSCO number scores, high sequence continuity (scaffold N50), and majority of sequences contained on the 30 pseudochromosomes (plus sex chromosome and mitochondrion). All in all, this is a valuable contribution by the Darwin Tree of Life.

Are sufficient details of methods and materials provided to allow replication by others?

Yes

Is the rationale for creating the dataset(s) clearly described?

Yes

Are the datasets clearly presented in a useable and accessible format?

Yes

Are the protocols appropriate and is the work technically sound?

Yes

Reviewer Expertise:

Genomics, evolution, invertebrates

I confirm that I have read this submission and believe that I have an appropriate level of expertise to confirm that it is of an acceptable scientific standard.

# Reviewer report: Reviewer response for version 1

**DOI:** 10.21956/wellcomeopenres.21683.r89249

**Published:** 2024-08-01

**Authors:** Elena Pazhenkova

**Affiliations:** 1 University of Ljubljana, Ljubljana, Slovenia

The study presents the first chromosome-level assembly for the Arctic Skipper, *Carterocephalus palaemon* (Lepidoptera, Hesperiidae). The karyotype for this species is not studied, but the assembly was assigned to 31 chromosomal pseudomolecules, and this chromosome number is known as an ancestral for the family Hesperiidae and the order Lepidoptera in general. Lukhtanov. (2014_ ^1^).

 The resulting assembly spans 394.5 Mb, sequencing was performed using PacBio single-molecule platform. Chromosome-scale scaffolds were confirmed by the Hi-C data, generated on the Illumina NovaSeq 6000 instrument.

 The article also provides an introduction, including data on species taxonomy, distribution, and conservation status. The genome assembly presented in this is an important contribution to the field of biodiversity research.

Are sufficient details of methods and materials provided to allow replication by others?

Yes

Is the rationale for creating the dataset(s) clearly described?

Yes

Are the datasets clearly presented in a useable and accessible format?

Yes

Are the protocols appropriate and is the work technically sound?

Yes

Reviewer Expertise:

phylogenetics, evolutionary biology, conservation biology, bioinformatics

I confirm that I have read this submission and believe that I have an appropriate level of expertise to confirm that it is of an acceptable scientific standard.

## References

[1] : Chromosome number evolution in skippers (Lepidoptera, Hesperiidae). *Comp Cytogenet* .2014;8(4) : 10.3897/CompCytogen.v8i4.8789 275-91 10.3897/CompCytogen.v8i4.8789 25610542 PMC4296715
